# Atmospheric Corrosion Behavior of Q235 Steel Exposed to the Subtropical Marine Environment in the East China Sea for Two Years

**DOI:** 10.3390/ma19061189

**Published:** 2026-03-18

**Authors:** Tianxing Chen, Lihui Yang, Cong Liu, Tianlong Zhang, Shibo Chen, Xiaoyan Deng, Liang Sun

**Affiliations:** 1 School of Environment and Safety Engineering, Qingdao University of Science and Technology, Qingdao 266042, China; 2 State Key Laboratory of Advanced Marine Materials, Institute of Oceanology, Chinese Academy of Sciences, Qingdao 266071, China; 3 Southwest Institute of Technology and Engineering, Chongqing 400039, China; 4 Qingdao Marine Science and Technology Center, Qingdao 266237, China

**Keywords:** subtropical marine atmosphere, carbon steel, atmospheric corrosion, corrosion behavior

## Abstract

The corrosion behavior and mechanism of Q235 steel during a two-year exposure to the subtropical marine atmospheric environment on an offshore platform in the East China Sea were investigated in this study. Methods including corrosion weight loss measurement, macro/micro-morphological observation (using a digital camera, SEM, and 3D-CLSM), composition analysis (XRD and XPS), and electrochemical tests (EIS and Tafel polarization curves) were employed to systematically examine corrosion kinetics, rust layer evolution, and electrochemical performance. The results indicated that the corrosion rate of Q235 steel initially increased and subsequently decreased with prolonged exposure, with the atmospheric corrosivity reaching CX level as defined (according to the ISO 9223 standard). The corrosion products transitioned from an early-stage rust layer predominantly consisting of γ-FeOOH to a later-stage layer primarily composed of α-FeOOH and Fe_3_O_4_. XPS analyses revealed that both the α*/γ* ratio and the Fe(II)/Fe(III) ratio increased over time, demonstrating a progressive improvement in the protective properties of the rust layer. The polarization resistance of the rust layer gradually rose, while the corrosion current density declined significantly, further confirming the enhanced stability and protective performance of the rust layer following long-term exposure. Chloride ions accumulated at defects within the rust layer, inducing local acidification, which played a key role in promoting the initiation and propagation of pitting corrosion. This study elucidated the corrosion behavior and mechanism of Q235 steel in the marine atmospheric environment of the East China Sea. Despite the increase in exposure time from 6 to 24 months, during which the electrochemical stability of the rust layer enhanced over time, it failed to prevent the initiation and propagation of severe localized corrosion—an issue of critical importance for load-bearing structures. The findings provide important theoretical and data support for service-life assessment and corrosion protection design of offshore photovoltaic steel structures.

## 1. Introduction

With the global shift towards a low-carbon energy structure, offshore photovoltaic (PV) power generation, as a renewable energy technology that utilizes vast ocean spaces and reduces land occupation, has attracted increasing attention. Compared to terrestrial environments, the marine environment offers higher PV module cooling efficiency and potential power generation gains [1,2,3]. However, the harsh marine corrosion environment—characterized by high salinity, high humidity, and intense ultraviolet radiation—poses severe threats to the structural integrity and operational safety of PV systems [4,5,6]. Among these challenges, the support structure, which serves as the mechanical backbone of the entire power station, directly determines the station’s lifespan, maintenance costs, and economic performance through the durability of its materials. Therefore, a deep understanding of the corrosion behavior and mechanism of offshore PV support structure materials in real marine environments is an indispensable scientific foundation for promoting the large-scale and commercial application of this technology.

Carbon steel, owing to its excellent mechanical properties, processability, and cost-effectiveness, has emerged as one of the primary candidate materials for offshore PV support structures [5]. Current research on the corrosion behavior of carbon steel in marine environments has yielded substantial results. It is widely recognized that its corrosion process is influenced by a combination of factors such as salinity, temperature, humidity, and light intensity, with corrosion kinetics typically following a power-law pattern [7,8,9]. For instance, Wang et al. investigated the corrosion behavior of carbon steel exposed for one year in the atmospheric environment of South China Sea, finding that the specimen surface underwent severe uniform corrosion under the harsh marine atmosphere [10]. Zhu et al. comprehensively studied the corrosion behavior of Q235 steel in the splash zone of Qingdao’s marine environment through field exposure tests and laboratory simulation experiments. Their study indicated that the corrosion behavior of Q235 steel is significantly affected by two factors: wet–dry ratio and splash frequency [11]. However, most existing studies have focused on the corrosion behavior of Q235 steel in simulated marine environments or coastal atmospheric zones. Systematic research tailored to the specific service conditions of offshore PV systems remains insufficient.

The East China Sea, as a key region for the development of offshore PV in China, possesses unique environmental conditions that may lead to distinct corrosion characteristics in steels compared to other sea areas. To quantitatively elucidate the unique corrosion environment of the East China Sea, we compared its key corrosion parameters with those of other typical marine environments in China (the South China Sea and Qingdao), as shown in Table 1. Table 1 reveals that the East China Sea exhibits a distinctive combination of environmental characteristics. The average temperature in the East China Sea is 20.5 °C, which lies between that of the South China Sea (28.8 °C) and Qingdao (12.3 °C). The average relative humidity in the East China Sea is 77.7%, which is essentially equivalent to that of the South China Sea (77.8%) and higher than that of Qingdao (72%). This high humidity condition facilitates the formation and persistence of an electrolyte layer on the steel surface, serving as a crucial factor in accelerating the electrochemical corrosion process. Regarding the chloride ion deposition rate, the East China Sea registers 81 mg/(m^2^·d), significantly higher than that of the temperate marine environment in Qingdao (16.6 mg/(m^2^·d)) but substantially lower than that of the tropical marine environment in the South China Sea (400 mg/(m^2^·d)). This suggests that its corrosion mechanism may differ from that of the South China Sea, which is dominated by extreme salt fog, thus establishing the East China Sea as a typical subtropical humid marine atmosphere environment. This combination of environmental parameters—”moderate temperature, high humidity, and intermediate salinity”—distinguishes the atmospheric corrosion environment of the East China Sea from those of other sea areas in China. This unique corrosive environment may lead to differences in the corrosion kinetics, rust layer structure, and electrochemical properties of carbon steel compared to those in other sea areas. However, research on the corrosion behavior of carbon steel during long-term outdoor exposure in this distinctive marine atmospheric environment of the East China Sea is currently lacking. This deficiency results in a lack of precise data and theoretical support for predicting the service life and designing protection strategies for materials used in offshore photovoltaic support structures in this region.

Against this background, this study focused on Q235 steel and conducted a 24-month field exposure test on a 26 m-high offshore platform in the East China Sea. The test simulated a critical environmental zone for offshore PV support structures: the marine atmospheric zone. This work systematically investigates the corrosion kinetics, rust layer morphology, compositional evolution, and electrochemical performance of Q235 steel exposed to the East China Sea marine environment for 24 months. Through this research, we aim to provide key data to support the long-term protection and safe service of PV steel structures in the East China Sea and similar marine environments, thereby contributing fundamental research insights to the large-scale development and sustainable advancement of offshore photovoltaics.

## 2. Experimental

### 2.1. Materials

In this study, the material used was Q235 steel, which was obtained from Shandong Yangxin County Shengxin Technology Co., Ltd. (Yangxin, Binzhou, China). Its delivery condition was hot-rolled, meaning it was supplied in its industrial-delivery state without any subsequent heat treatment. Its chemical composition is shown in Table 2. The sample dimensions were 100 mm × 50 mm × 3 mm. All specimens were ground with 800-grit sandpaper, cleaned with acetone to remove surface contaminants, dried in flowing air, and then weighed to determine their initial mass. The samples were retrieved after outdoor exposure for 6, 12, and 24 months, respectively, with five parallel specimens collected for each exposure period. Among these, three specimens were used for analyzing weight loss and observing corrosion morphology. The remaining two parallel specimens were, respectively, used for characterization of the corrosion products and electrochemical analysis.

### 2.2. Field Exposure Tests

**Figure 1:**
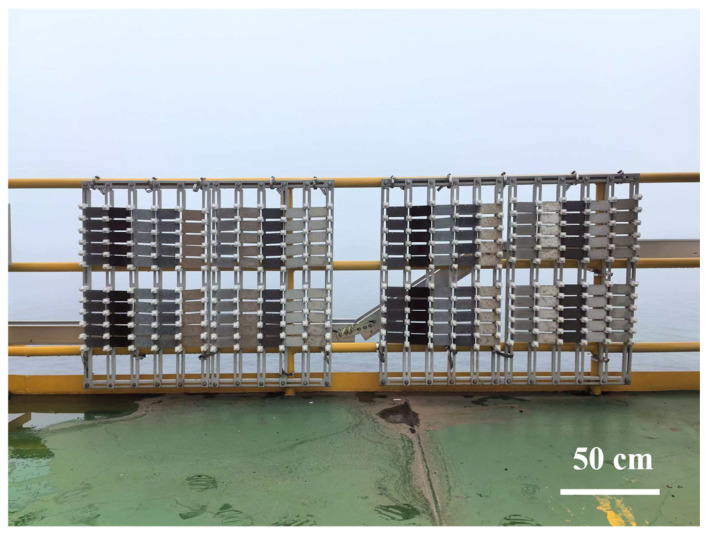
Setup of Q235 Steel exposed at 26 m offshore platform in the East China Sea.

The field exposure test site was located on a 26 m-high offshore platform in the East China Sea, as shown in Figure 1. The region experiences a typical subtropical maritime climate characterized by high humidity, high salt spray, intense solar radiation, and abundant precipitation. The average temperature is 20.5 °C, the average relative humidity is 77.7%, the chloride deposition rate is 81 mg/(m^2^·d), and the time of wetness (TOW) is 4022 h. The atmospheric corrosivity reaching CX level is as defined (according to the ISO 9223 standard [14]). In accordance with the ISO 8565:1992 standard [15], the filed exposure specimens were mounted facing south at a 45° angle to the horizontal plane on atmospheric corrosion test racks for a total test period of two years.

### 2.3. Determination of Corrosion Rates

In accordance with the procedure specified in ISO 9226:2012 [16], a derusting solution was prepared and used to remove corrosion products from the surface of Q235 steel specimens exposed for various periods. The samples were subsequently rinsed sequentially with deionized water and ethanol, followed by drying in flowing air. Finally, the mass of the substrate after corrosion product removal was measured. The corrosion rate was calculated using Equation (1) [16]:

(1) $$r_{corr}\;=\;\frac{∆m}{A\cdot \rho \cdot t}$$

where *r_corr_* is the corrosion rate (µm/a), ∆*m* is the mass loss (g), *A* is the surface area (m^2^), *ρ* is the density (7.85 g/cm^3^), and *t* is the exposure time (a).

### 2.4. Analysis of Corrosion Products

Macroscopic morphologies of the rust layer formed on the Q235 steel surface and the substrate after the removal of corrosion products were captured using a digital camera. The microscopic morphologies of the corrosion products, the cross-sectional microstructures of the rust layer, as well as the elemental composition and distribution, were examined using a scanning electron microscope (SEM, Zeiss Sigma500, Oberkochen, Germany) at an accelerating voltage of 3 kV and a working distance of 5.7 mm. The microscopic morphology of the substrate after corrosion product removal and the depth of corrosion pits were measured using a 3D laser scanning confocal microscope (3D-CLSM, Olympus OLS4100, Shinjuku, Japan). Phase composition of the corrosion products was analyzed by X-ray diffraction (XRD, PANalytical X-Pert3 Powder, Malvern Panalytical, Almelo, The Netherlands) with a Co target at 40 kV and 40 mA. The scanning range was 5–85° with a speed of 8°/min. Elemental composition and chemical states of the corrosion products were further characterized by X-ray photoelectron spectroscopy (XPS, Thermo Fisher Scientific Nexsa, Waltham, MA, USA) with an Al Kα X-ray source and a pass energy of 30 eV. The binding energies of the XPS spectra were calibrated using the C 1 s peak (284.8 eV) as a reference. The peaks were fitted using the Shirley background mode.

### 2.5. Electrochemical Test

The electrochemical impedance spectroscopy (EIS) of the corrosion products was measured using an electrochemical workstation (Princeton PARSTAT P4000+, Oak Ridge, TN, USA). A standard three-electrode system was employed, with the rusted Q235 steel (exposing an area of 1 cm^2^) as the working electrode, an Ag/AgCl electrode as the reference electrode, and a platinum mesh as the counter electrode. The electrolyte was a 3.5 wt.% NaCl aqueous solution. Prior to the EIS measurement, the open circuit potential (OCP) was monitored for 1 h to reach a stable state. The EIS was then performed at a potential of OCP with a frequency range from 10^5^ Hz to 10^−2^ Hz and an AC amplitude of 10 mV. Finally, Tafel polarization curves were measured at a scan rate of 0.33 mV/s within the potential range of −0.5 V to +0.5 V (vs. OCP).

## 3. Results and Discussion

### 3.1. Corrosion Kinetics

**Figure 2:**
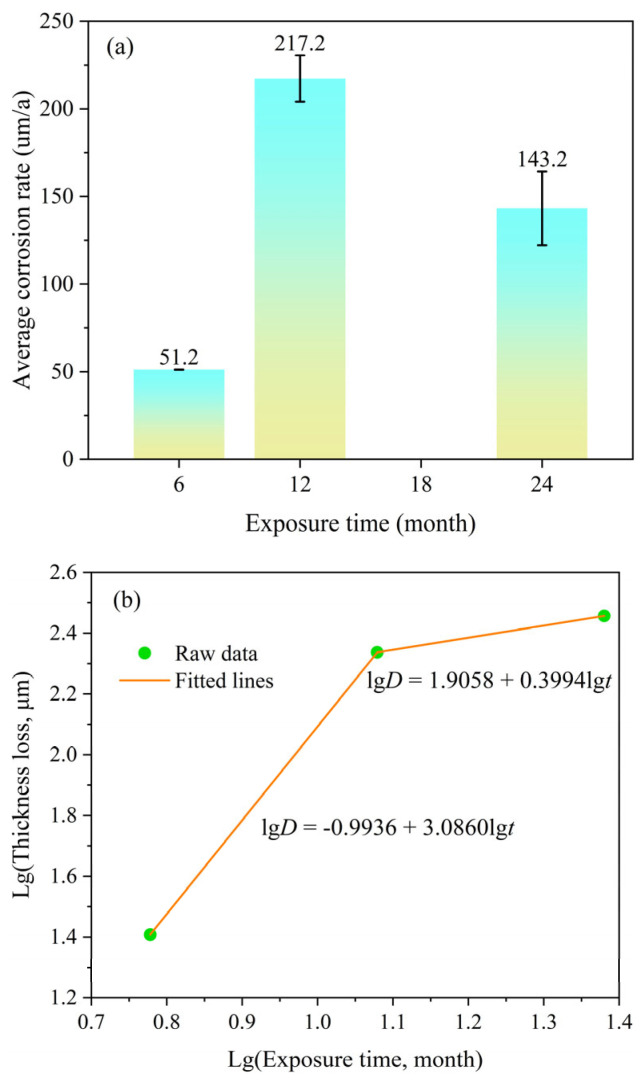
(**a**) Corrosion rate and (**b**) thickness loss of Q235 steel in the East China Sea versus exposure time.

Figure 2a shows the variation in corrosion rate of Q235 steel with exposure time in the East China Sea. The corrosion rate initially increases and then decreases over time, exhibiting significant overall fluctuation. After 6 months of exposure, the corrosion rate was 51.2 µm/a, which increased to 217.2 µm/a at 12 months, and subsequently decreased to 143.2 µm/a after 24 months of exposure. Townsend and Zoccola [17] proposed a method for analyzing corrosion data using linear regression, which involves fitting a straight line on a plot of logarithmic rust layer mass loss versus logarithmic exposure time. This approach can be used to predict the corrosion depth of steel over longer exposure periods. The evolution of atmospheric corrosion kinetics for Q235 steel follows Equation (2):

lg*D* = lgK + nlg*t* (2)

where *D* is the thickness loss (μm); K is a constant; n is a constant related to the protective properties of the corrosion products—n < 1 indicates a gradually decreasing corrosion rate, suggesting that the corrosion products are protective; n = 1 indicates a constant corrosion rate; and n > 1 indicates a gradually increasing corrosion rate, suggesting that the corrosion products lack protective properties; *t* is the exposure time. The trend of the three data points in Figure 2b preliminarily suggests that the corrosion kinetics may have changed over time. The slope between the first two points appears steeper than that between the last two points. Although the limited number of exposure periods precludes a definitive segmented regression analysis, a preliminary fit of these two segments indicates that n > 1 for the first segment and n < 1 for the second segment [18]. This trend tentatively suggests that the corrosion products in the initial stage may not be protective for the substrate, leading to an increase in the corrosion rate. However, after 12 months, the rust layer may gradually become more protective, resulting in a decrease in the corrosion rate. Additional data points at more exposure times are needed to confirm this two-stage corrosion behavior.

### 3.2. Morphology Observation

**Figure 3:**
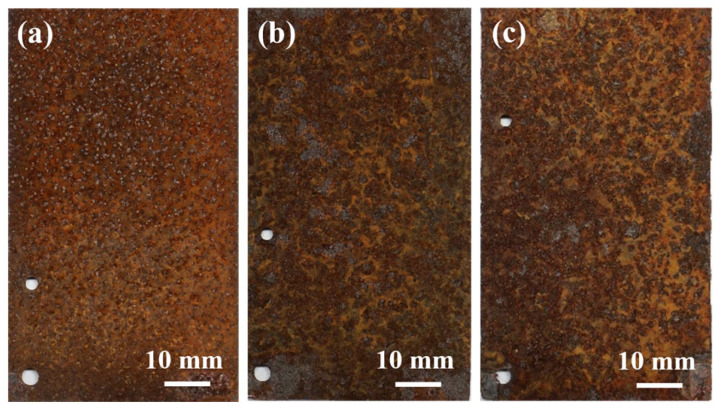
Macroscopic morphology of rust layer on Q235 steel after exposure for different periods: (**a**) 6 months, (**b**) 12 months, and (**c**) 24 months.

Figure 3 illustrates the evolution of the macroscopic morphology of the rust layers formed on Q235 steel after different exposure periods. After 6 months of exposure, a relatively uniform and thin rust layer was formed on the Q235 steel surface. The layer exhibited overall integrity with no significant signs of spalling, and its color was predominantly reddish-brown. After 12 months of exposure, the morphology of the rust layer underwent significant changes. The rust layer became noticeably thicker, its structure turned porous, and localized spalling began to appear. Yellowish-brown areas emerged on the surface, indicating distinct changes in the phase composition of the corrosion products. After 24 months of exposure, significant flaking and spalling occurred at the edges of the rust layer. The extent of yellowish-brown areas on the surface further expanded, suggesting continuous alterations in the phase composition of the corrosion products.

**Figure 4:**
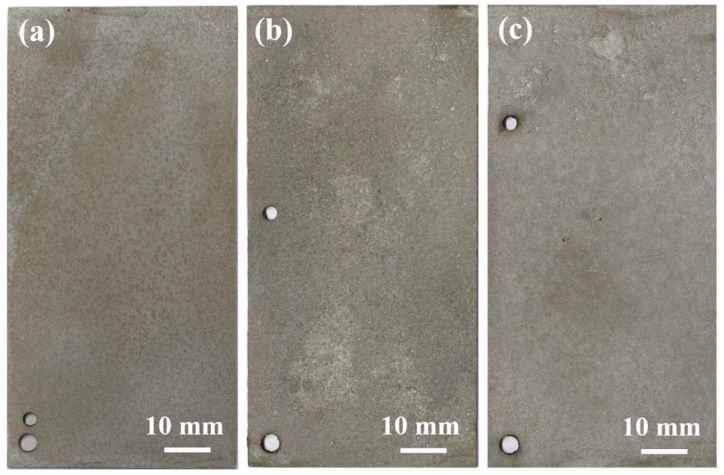
Macroscopic morphology of the substrate of Q235 steel after removal of corrosion products at different exposure time: (**a**) 6 months, (**b**) 12 months, and (**c**) 24 months.

Figure 4 illustrates the evolution of the macroscopic morphology of the Q235 steel substrate after the removal of corrosion products following different exposure periods. After 6 months of exposure, the substrate surface exhibited numerous, relatively uniformly distributed pits, with overall shallow corrosion depths. After 12 months of exposure, the corrosion morphology underwent significant changes due to the non-uniformity of the rust layer structure and variations in the local electrochemical environment. The initially formed isolated pits gradually interconnected and merged, evolving into groove-like or patch-like corrosion regions with notably increased width and depth. This transformation indicates a shift in the corrosion mode from uniformly distributed pitting to non-uniform localized corrosion. After 24 months of exposure, corrosion intensified further, forming a continuous, deeper and wider corrosion region, particularly evident in the upper-right area. This resulted in a noticeable thinning of the substrate material in that region.

**Figure 5:**
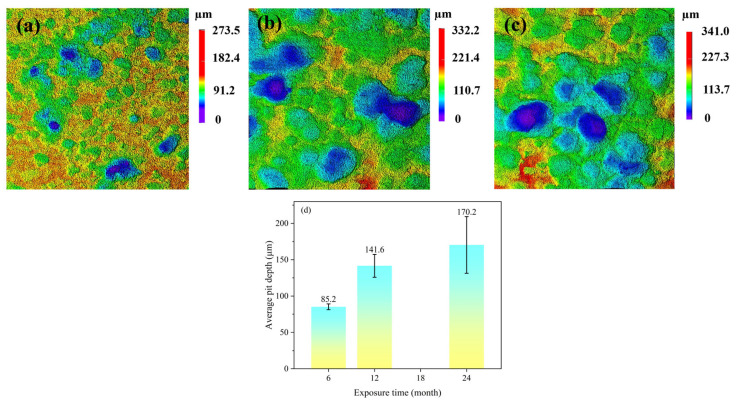
3D laser confocal microscopy images of the Q235 steel substrate after removal of corrosion products at various exposure time: (**a**) 6 months, (**b**) 12 months, (**c**) 24 months and (**d**) the corresponding average pit depth. Note: Localized corrosion of Q235 at various exposure times was evaluated using three specimens. For each test surface, the five deepest corrosion pits were measured. The average depth of all measured points is reported as the mean pit depth, and the maximum value among them is reported as the maximum pit depth.

Figure 5 presents the 3D laser scanning confocal microscopy images of the Q235 steel substrate after the removal of corrosion products following different exposure periods. After 6 months of exposure, the primary form of corrosion was general corrosion, characterized by a relatively flat and smooth surface, accompanied by a small number of localized corrosion pits. To quantitatively evaluate the localized corrosion, a statistical analysis of the corrosion pits on the specimens was conducted. For the specimen exposed for 6 months, the results showed a mean pit depth of 85.2 μm, a maximum pit depth of 89.791 μm, and a standard deviation of 5.0. When the exposure time was extended to 12 months, the localized corrosion phenomenon intensified significantly, with increases in both the width and depth of the corrosion pits. Analysis of the 12-month specimen revealed a mean pit depth of 141.6 μm, a maximum pit depth of 158.542 μm, and a standard deviation of 15.8. For the specimen exposed for 24 months, the results showed a mean pit depth of 170.2 μm, a maximum pit depth of 213.352 μm, and a standard deviation of 38.9. The increases in the mean pit depth, maximum pit depth, and standard deviation demonstrate that as exposure time progresses, the localized corrosion becomes increasingly severe, and its heterogeneity continues to grow. This transition from general to localized corrosion can be attributed to the non-uniformity of the corrosion product layer, the continuous penetration of corrosive media such as chloride ions, and the prolonged action of galvanic couples [19].

**Figure 6:**
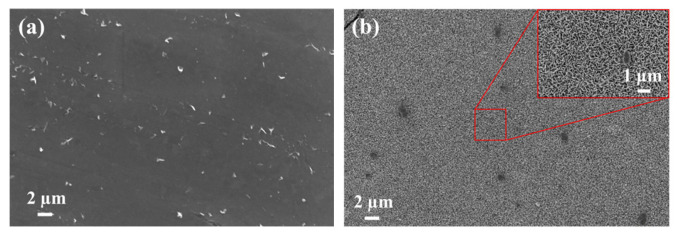

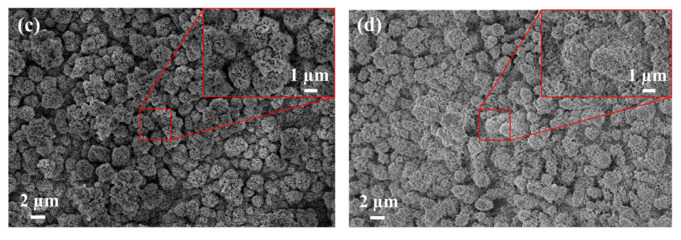
Surface microscopic morphology of rust layers on Q235 steel with different exposure time: (**a**) 0 months, (**b**) 6 months, (**c**) 12 months, and (**d**) 24 months.

Figure 6 presents the surface micromorphology of the rust layers formed on Q235 steel after different exposure periods. The surface of the Q235 steel substrate before exposure exhibits parallel grinding marks and a clean morphology, with no obvious corrosion products observed. Studies indicate that the morphology of γ-FeOOH (lepidocrocite) can be primarily categorized into two types: flake-like and spherical [20,21]. After 6 months of exposure, the rust layer surface mainly exhibited a stacked arrangement of flake-like γ-FeOOH structures. These flakes were interlocked and layered, forming a relatively loose surface morphology. As exposure time extended to 12 months, the morphology showed a significant change. At this stage, γ-FeOOH primarily appeared in the form of spherical particles that aggregated [22]. These spherical particles were densely packed, with clusters observed in certain areas. When exposure time further increased to 24 months, a notable transformation occurred in the rust layer phases, with α-FeOOH (goethite) becoming the predominant corrosion product. Its morphology displayed a characteristic dense granular structure [23], indicating that the rust layer gradually stabilized over time. This dense and stable rust layer provides a degree of protection to the substrate, slowing down further corrosion progression.

**Figure 7:**
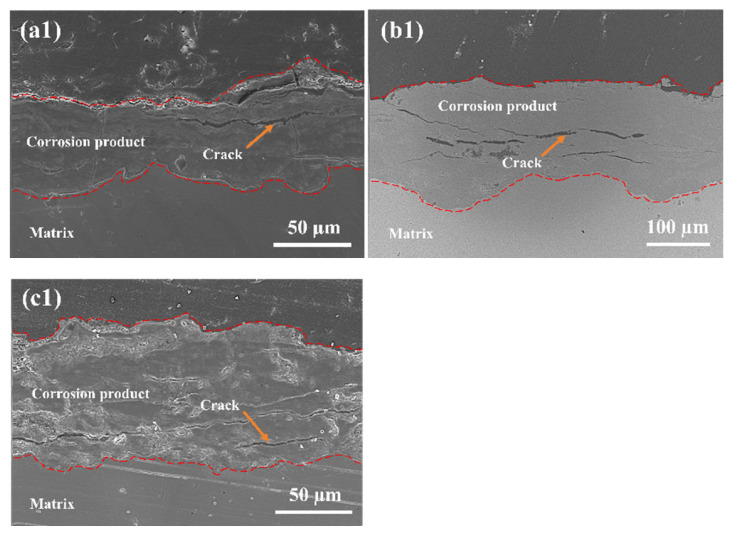

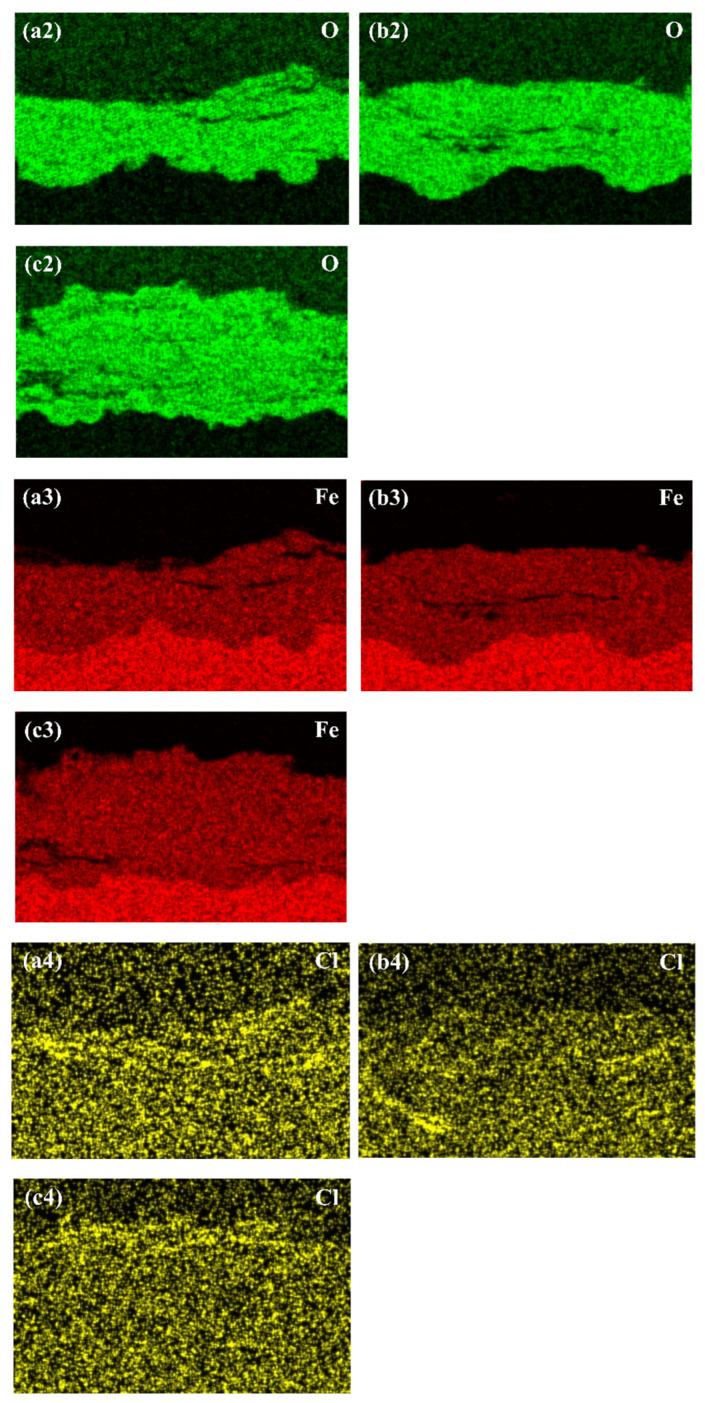
Cross-sectional microstructures and corresponding elemental mapping of rust layers on Q235 steel with varying exposure time: (**a1**–**a4**) 6 months, (**b1**–**b4**) 12 months, and (**c1**–**c4**) 24 months.

Figure 7 presents the cross-sectional micromorphology and corresponding elemental distribution of the rust layers formed on Q235 steel after different exposure periods. Over the two-year exposure duration, significant changes were observed in the cross-sectional morphology of the rust layer. After 6 months of exposure, the rust layer had a thickness of approximately 62 μm. Its structure was relatively dense with few cracks, and the interface between the rust layer and the substrate was distinct. Following 12 months of exposure, the rust layer significantly thickened to about 180 μm, accompanied by a marked increase in the number of internal cracks. These cracks progressively propagated and penetrated towards the rust layer–substrate interface. This structural change facilitated the further infiltration of aggressive media, such as Cl^−^. Detection results confirmed that Cl^−^ had permeated to the rust–substrate interface, which would accelerate the corrosion process at this interface. After 24 months of exposure, the rust layer thickness decreased to approximately 85 μm, noticeably thinner compared to the 12-month sample. This reduction was attributed to the further propagation and interconnection of internal cracks within the rust layer, leading to localized spalling. Elemental distribution maps reveal that the distribution range of oxygen within the rust layer gradually expanded with increasing exposure time, further corroborating the ongoing corrosion development and structural evolution of the rust layer. These results demonstrate that with prolonged exposure, the rust layer on Q235 steel undergoes a process of thickening, cracking, infiltration of corrosive media, and eventual spalling. These structural changes directly influence the protective performance of the rust layer and the corrosion rate of the steel.

### 3.3. Composition Analysis of Corrosion Products

**Figure 8:**
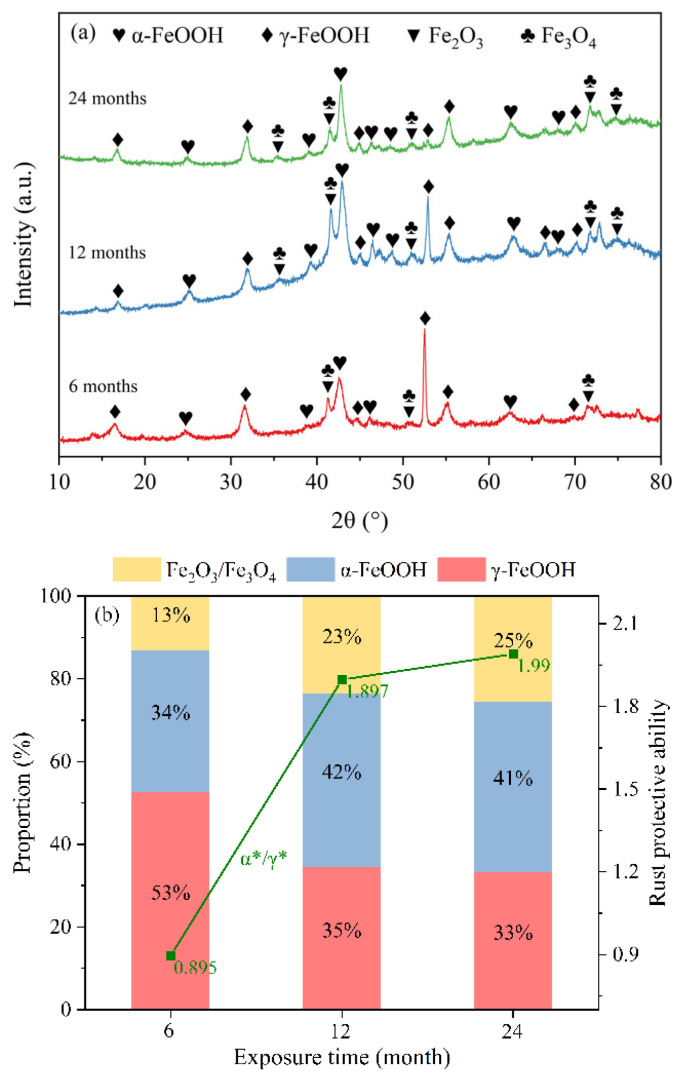
(**a**) XRD patterns of rust layers on Q235 steel at different exposure durations and (**b**) semi-quantitative analysis result of the XRD data in the rust layers.

Figure 8a shows the XRD patterns of the rust layers formed on Q235 steel under different exposure times. As can be seen from the figure, the rust layers of Q235 steel at different exposure durations are all composed of α-FeOOH, γ-FeOOH, Fe_2_O_3_, and Fe_3_O_4_. β-FeOOH tends to undergo electrochemical reduction and is the iron oxyhydroxide most likely to transform into Fe_3_O_4_. When exposed to environments with high chloride concentrations and high humidity, β-FeOOH converts to Fe_3_O_4_, with the transformation propensity following the order: β-FeOOH > γ-FeOOH > α-FeOOH [24,25]. This finding supports the absence of β-FeOOH in corrosion products and is consistent with the studies by Niu and Wang [26,27]. Previous studies have indicated that the α*/γ* ratio can serve as an indicator for evaluating the stability and protective performance of the rust layer, defined as (α + S)/(γ + β), where S represents the total content of Fe_2_O_3_ and Fe_3_O_4_ [28,29]. As shown in Figure 8(b), the semi-quantitative analysis results reveal that the order of the α*/γ* ratio is: 24 months > 12 months > 6 months. This trend suggests that the protective performance of the rust layer gradually improves with prolonged exposure time.

**Figure 9:**
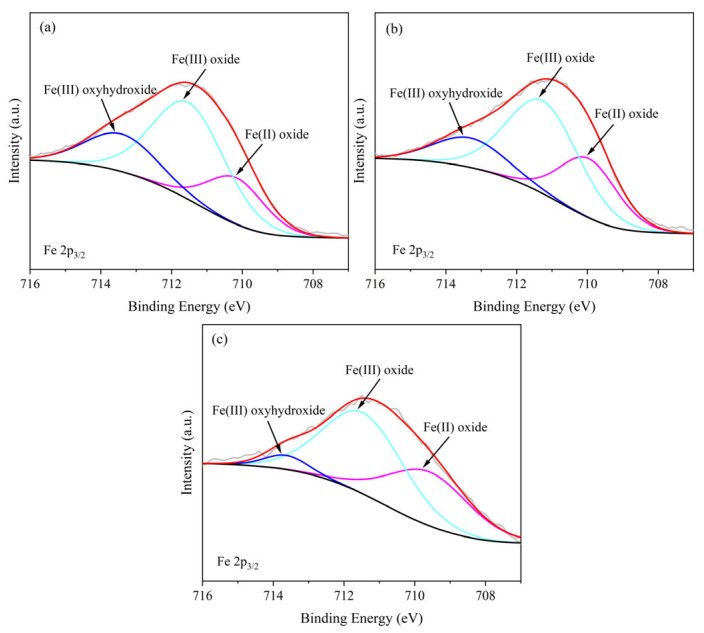
High-resolution XPS spectra of Fe 2p_3/2_ in rust layers of Q235 steel under different exposure time: (**a**) 6 months, (**b**) 12 months, and (**c**) 24 months.

To gain a more accurate understanding of the evolution of the chemical composition of corrosion products on Q235 steel exposed to the marine atmospheric environment of the East China Sea, XPS characterization was performed on the rust layers. The results are presented in Figure 9. The high-resolution XPS spectra of Fe 2p_3/2_ reveal three distinct characteristic peaks, corresponding to Fe(II) oxides, Fe(III) oxides, and Fe(III) oxyhydroxides, respectively [30,31]. Table 3 summarizes the parameters of the characteristic peaks from the high-resolution Fe 2p_3/2_ XPS spectra for the rust layers at different exposure times. The data indicate that Fe(III) oxides remain the predominant iron species within the rust layer, regardless of exposure duration. According to previous studies, Fe(II) oxides in steel corrosion products exhibit greater corrosion resistance compared to Fe(III) oxides [32,33]. Consequently, the relative content ratio of Fe(II)/Fe(III) can serve as a key indicator for evaluating the protective performance of the rust layer: a higher ratio suggests a more stable structure, better corrosion resistance, and stronger protective capability for the substrate. Analysis of the variation trend in the Fe(II)/Fe(III) ratio across different exposure durations reveals that this ratio increases progressively with prolonged exposure. This trend indicates that during long-term exposure, the rust layer undergoes continuous phase transformation. The proportion of the more stable and corrosion-resistant Fe(II) oxides increases, signifying an evolution of the rust layer towards a more protective structure.

### 3.4. Electrochemical Analysis

**Figure 10:**
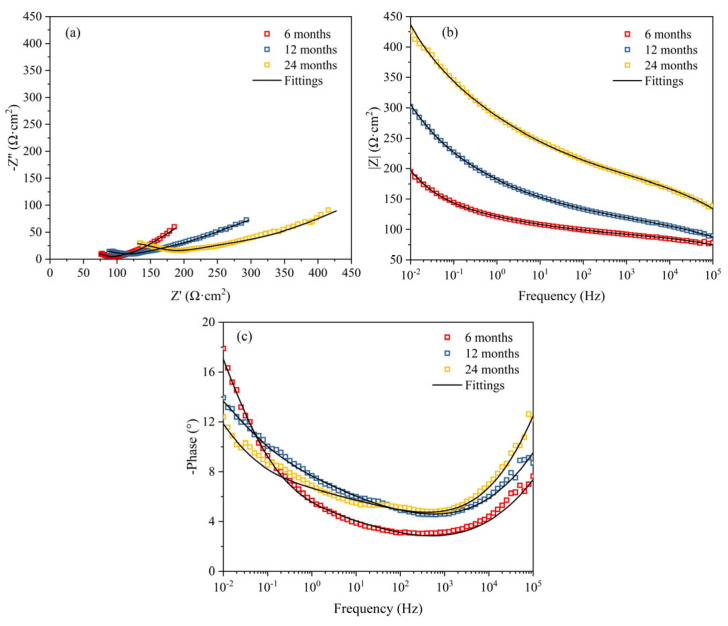
EIS of Q235 steel under different exposure time: (**a**) Nyquist plots, (**b**) Bode-|Z| plots, and (**c**) Bode-Phase plots.

**Figure 11:**
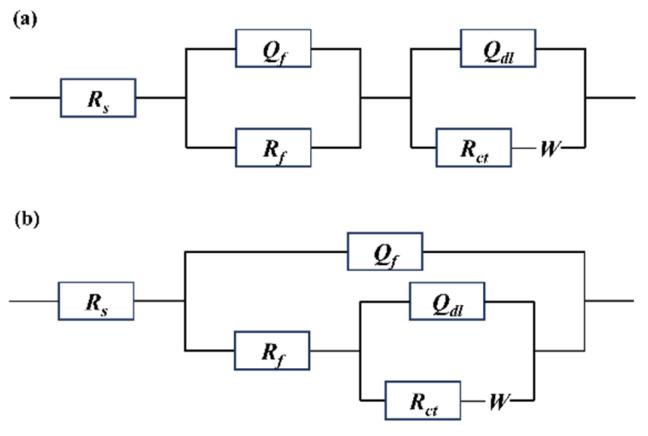
Equivalent circuits for fitting EIS data of Q235 steel at different exposure periods: (**a**) for 6 and 24 months; (**b**) for 12 months.

EIS is an effective method for investigating the protective performance of corrosion product films on steel surfaces [34,35]. Therefore, EIS measurements were conducted on Q235 steel after different exposure periods, as shown in Figure 10. The data were fitted using the equivalent circuits depicted in Figure 11. Specifically, the EIS data for samples exposed for 6 and 24 months were fitted using the equivalent circuit shown in Figure 11a, while the data for the 12-month sample were fitted using the circuit in Figure 11b. These circuits include solution resistance (*R_s_*), rust layer capacitance (*Q_f_*), rust layer resistance (*R_f_*), double-layer capacitance (*Q_dl_*), charge transfer resistance (*R_ct_*), and Warburg impedance (*W*). The fitted parameters of the equivalent circuits are summarized in Table 4. At 6 months of exposure, the rust layer is relatively dense with few cracks, and the rust layer/substrate interface remains intact. At 24 months of exposure, the outer rust layer becomes loose and spalls off, leading to a reduction in rust layer thickness, although the remaining rust layer remains relatively dense and provides a protective barrier. In both cases, ions must penetrate the rust layer before reaching the substrate, and thus the time constants exhibit a series characteristic (Figure 11a). At 12 months of exposure, the rust layer thickens significantly, with a substantial increase in internal cracks that extend to the interface. Cl^−^ ions penetrate directly to the substrate surface through these cracks, forming localized conductive pathways. At this stage, the impedance of the rust layer and the charge transfer process at the substrate occur simultaneously, making a parallel circuit more suitable for fitting (Figure 11b). The polarization resistance (*R_p_*), defined as *R_p_* = *R_f_* + *R_ct_*, serves as a comprehensive indicator for evaluating the protective capability of the rust layer on the substrate. A higher *R_p_* value signifies better protective performance of the rust layer [36,37]. As shown in Table 3, the *R_p_* increases markedly as the exposure time extends from 6 to 12 months. However, with further exposure to 24 months, the *R_p_* value remains relatively stable, indicating that the protective performance of the rust layer reaches a steady state after one year of atmospheric exposure. Furthermore, according to previous studies, the magnitude of the impedance modulus at low frequency (|Z|_0.01Hz_) can also be used for qualitative analysis of the rust layer’s protective performance [38,39]. Observing the variation of |Z|_0.01Hz_ with exposure time in Figure 10b, its value is found to increase progressively. This trend further confirms that the protective capability and stability of the rust layer are enhanced with increasing exposure time.

**Figure 12:**
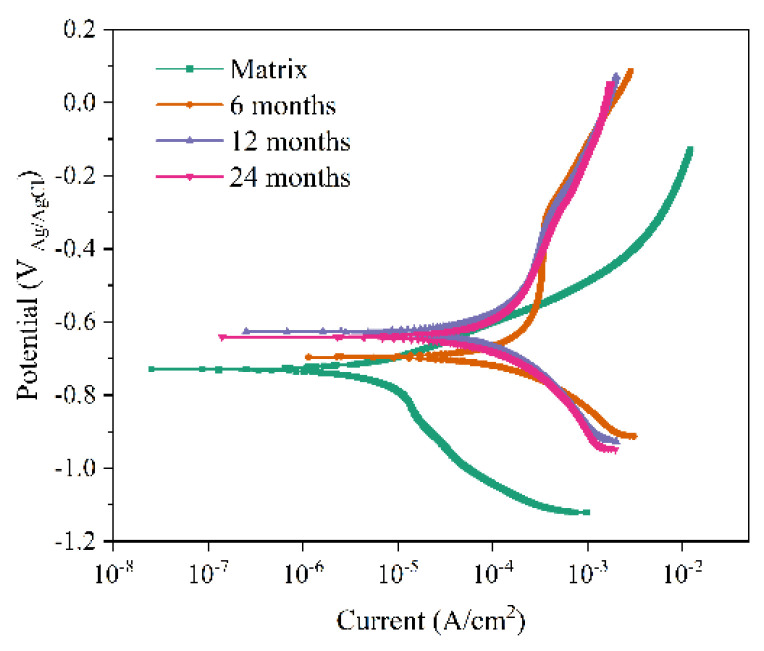
Tafel polarization curves of Q235 steel under different exposure time.

Figure 12 presents the Tafel polarization curves of Q235 steel after different exposure periods. As observed, the cathodic branches of the polarization curves at various exposure times exhibited similar characteristics, suggesting that the corrosion products formed on the Q235 steel surface have a relatively limited influence on the cathodic reaction process. For bare Q235 steel without a rust layer, its cathodic process is primarily controlled by the reduction in dissolved oxygen, while the anodic process is governed by the dissolution of the substrate itself. However, after the formation of a rust layer, the cathodic process involves both oxygen reduction and rust layer reduction, with the latter becoming dominant [40]. Notably, the polarization curve for the sample exposed for 6 months shows a relatively wide passivation region in its anodic branch, and a distinct pitting potential is observed. When the anodic potential exceeds this value, the current density increases sharply, indicating the breakdown of the passive film. This phenomenon suggests that the corrosion product layer at this stage provides a certain degree of protection to the substrate [41]. The corrosion potential (*E*_corr_) and corrosion current density (*I*_corr_) for Q235 steel at different exposure times were obtained through linear extrapolation, and the specific data are listed in Table 5. Generally, *I*_corr_ is a key parameter for evaluating the corrosion resistance of a rust layer [42]. The *I*_corr_ of the Q235 steel substrate was significantly lower than that of the sample exposed for 6 months. This phenomenon indicates that the rust layer formed on the Q235 steel surface after 6 months of exposure fails to provide effective protection and instead accelerates the corrosion of the substrate. When the exposure time was extended from 6 to 24 months, the Icorr gradually decreased from 3.7375 × 10^−5^ A/cm^2^ to 2.5150 × 10^−6^ A/cm^2^. From the perspective of corrosion kinetics, this trend indicates that the corrosion resistance of the rust layer improves with increasing exposure time. This finding aligns with the results obtained from the earlier XPS analysis.

However, it is worth noting that the enhanced electrochemical performance of the rust layer does not effectively prevent severe localized corrosion, and deep pitting corrosion still occurs, driven by chloride penetration through structural defects, which is particularly important for load-bearing structures in offshore photovoltaic applications.

### 3.5. Corrosion Mechanism

Corrosion kinetics results indicated that the corrosion rate decreased after 12 months. Combined with XRD and SEM results, this was attributed to the increased content of protective α-FeOOH and Fe_3_O_4_ within the rust layer. These dense phases reduced the porosity of the rust layer, hindering the transport of charge and corrosive media, thereby decreasing the corrosion rate. Meanwhile, XPS analysis results showed a continuously increasing Fe(II)/Fe(III) ratio, indicating the enrichment of more corrosion-resistant iron oxides. This chemical change directly led to an alteration in the electrochemical behavior of the rust layer. The *R_p_* increased significantly from 91.13 Ω·cm^2^ to 663.4 Ω·cm^2^, while the *I*_corr_ gradually decreased from 3.7375 × 10^−5^ A/cm^2^ to 2.5150 × 10^−6^ A/cm^2^. This confirms the intrinsic relationship between the chemical stability of the rust layer and its electrochemical protective performance. Although the electrochemical tests demonstrated continuously enhanced electrochemical performance of the rust layer, macroscopic and microscopic morphology revealed severe localized corrosion, with the average pit depth increasing from 85.2 μm to 170.2 μm. This seemingly contradictory phenomenon can be explained by the role of Cl^−^. Combined with mapping results, Cl^−^ penetrated and accumulated through defects in the rust layer, undergoing hydrolysis reactions that led to a localized pH decrease and the formation of an acidified environment, thereby initiating and promoting the propagation of localized corrosion. The rust layer becomes more electrochemically stable, but does not effectively limit localized corrosion.

Based on the above analysis, the corrosion mechanism of Q235 steel in the marine atmospheric environment of the East China Sea can be described as follows. The marine atmospheric environment, characterized by high humidity and high Cl^−^ content, constitutes a harsh electrochemical corrosion environment. In this setting, the corrosion of Q235 steel is driven by a typical electrochemical corrosion process involving a corrosion microcell composed of a thin surface electrolyte film, the metal substrate, and oxygen. The specific reactions are represented by Equations (3) and (4) [43,44]. At this stage, the corrosion is in its initial phase, with corrosion products covering a limited area and forming a relatively thin layer.

Anode: Fe → Fe^2+^ + 2e^−^ (3)

Cathode: 2H_2_O + O_2_ + 4e^−^ → 4OH^−^ (4)

At the anodic region, Fe^2+^ generated from Q235 steel reacts with OH^−^ produced at the cathodic region to form Fe(OH)_2_ (Equation 5):

Fe^2+^ + 2OH^−^ → Fe(OH)_2_ (5)

Fe(OH)_2_ is subsequently oxidized into metastable γ-FeOOH, which then undergoes phase transformation to form thermodynamically stable α-FeOOH or dissolves to produce Fe_2_O_3_ (Equations (6)–(8)):

4Fe(OH)_2_ + O_2_ → 4γ-FeOOH + 2H_2_O (6)

γ-FeOOH → α-FeOOH(7)

2γ-FeOOH → Fe_2_O_3_ + H_2_O (8)

In the final stage of rust layer evolution, FeOOH can be reduced to Fe_3_O_4_ by electrons generated from substrate dissolution or by Fe^2+^ in a humid environment (Equations (9) and (10)) [11,45]:

3FeOOH + H^+^ + e^−^ → Fe_3_O_4_ + 2H_2_O (9)

2FeOOH + Fe^2+^ → Fe_3_O_4_ + 2H^+^ (10)

In environments with high chloride ion concentrations, chloride ions play a catalytic role in the corrosion of Q235 steel (Equations (11) and (12)) [46]:

Fe^2+^ + 2Cl^−^ → FeCl_2_ (11)

FeCl_2_ + 2OH^−^ → Fe(OH)_2_ + 2Cl^−^ (12)

O_2_ can continuously oxidize Fe^2+^ to Fe^3+^. As a highly reactive and corrosive anion, Cl^−^ possesses strong penetrating ability and tends to migrate preferentially from defects in the surface rust layer to the substrate. At the metal–rust interface, Cl^−^ not only induces stress concentration and promotes crack formation [47] but also accumulates and catalyzes the hydrolysis of Fe^3+^, leading to the formation of FeOOH (Equation 13). This process is accompanied by the development of localized acidic conditions, further facilitating the initiation and propagation of pitting corrosion.

Fe^3+^ + 2H_2_O → FeOOH + 3H^+^ (13)

With prolonged exposure time, the initial corrosion products gradually thicken and extend into adjacent areas. Through continuous growth and diffusion of corrosion products, multiple corrosion sites progressively merge in three-dimensional space, leading to further expansion of the corroded regions and the formation of a continuous rust layer structure.

## 4. Conclusions

This study investigated the corrosion behavior and mechanism of Q235 steel after 6, 12, and 24 months of exposure to the marine atmosphere of the East China Sea. The main conclusions are as follows:

(1) The corrosion rate of Q235 steel first increases and then decreases. A two-stage corrosion kinetic model was proposed. In the first stage, n > 1, indicating that the corrosion products offer no protective effect on the substrate. In the second stage, n < 1, suggesting that the corrosion products provide protection to the substrate.

(2) With increasing exposure time, the rust layer evolved both morphologically and compositionally, and XRD and XPS analyses confirmed a continuous increase in the α*/γ* ratio and Fe(II)/Fe(III) ratio.

(3) Electrochemical measurements further confirmed the enhanced corrosion resistance of the rust layer, as evidenced by a significant increase and subsequent stabilization of *R_p_*, alongside a continuous rise in |Z|_0.01Hz_ and a decrease in corrosion current density over the two-year exposure period.

(4) Despite the continuous improvement in electrochemical performance, the rust layer failed to prevent the initiation and propagation of localized corrosion. After 24 months of exposure, the average pit depth reached 170.2 μm, accompanied by a significant thinning of the rust layer. Chloride ions penetrated through structural defects, promoting localized acidification and the development of pitting corrosion.

(5) These findings provide important theoretical and data support for the service life assessment and corrosion protection design of offshore photovoltaic support structure materials. Future research should integrate atmospheric corrosion monitoring techniques to enable continuous tracking of the corrosion process and deepen the understanding of the dynamic corrosion behavior under marine atmospheric conditions.

## Figures and Tables

**Table 1:** Environmental parameters of different marine atmospheres.

Environmental Parameter	East China Sea [This Study]	South China Sea [12]	Qingdao [13]
**Average temperature** **(°C)**	20.5	28.8	12.3
**Average relative humidity** **(%)**	77.7	77.8	72
**Chloride deposition rate** **(mg/(m^2^·d))**	81	400	16.6

**Table 2:** Chemical compositions of Q235 steel (mass fraction/%).

C	Si	Mn	P	S	Fe
0.17	0.21	0.43	0.019	0.023	Balance

**Table 3:** Parameters of characteristic peaks in high-resolution XPS spectra of Fe 2p_3/2_ for rust layers on Q235 steel under different exposure time (BE: Binding energy).

Peak Assignment	6 Months			12 Months			24 Months		
	Peak BE (eV)	Atomic Intensity (%)	Fe(II)/Fe(III)	Peak BE (eV)	Atomic Intensity (%)	Fe(II)/Fe(III)	Peak BE (eV)	Atomic Intensity (%)	Fe(II)/Fe(III)
Fe(II) oxide	710.18	21.70	0.38	709.96	27.92	0.51	709.89	37.81	0.67
Fe(III) oxide	711.51	56.41		711.26	54.62		711.46	56.84	
Fe(III) oxyhydroxide	713.41	21.90		713.31	17.46		713.63	5.35	

**Table 4:** The fitting results of EIS for Q235 steel at different exposure time.

Exposure Time/(Month)	Y_f_/(Ω^−1^·cm^−2^·s^n^)	n_f_	*R_f_*/(Ω·cm^2^)	Y_dl_/(Ω^−1^·cm^−2^·s^n^)	n_dl_	*R*_ct_/(Ω·cm^2^)
6	2.099 × 10^−5^	0.3717	91.13	0.0107	0.3219	69.45
12	1.804 × 10^−5^	0.3832	108.2	0.0076	0.2203	549.5
24	5.649 × 10^−6^	0.4951	159.4	0.0040	0.2072	504

**Table 5:** *E*corr and *I*corr of Q235 steel under different exposure time.

Exposure Time/(Month)	*E*corr (V)	*I*corr (A/cm^2^)
0	−0.6843	2.4056 × 10^−6^
6	−0.7260	3.7375 × 10^−5^
12	−0.6426	5.6385 × 10^−6^
24	−0.6585	2.5150 × 10^−6^

## Data Availability

The original contributions presented in this study are included in the article. Further inquiries can be directed to the corresponding authors.
